# Detection of the Lychee Erinose Mite, *Aceria litchii* (Keifer) (Acari: Eriophyidae) in Florida, USA: A Comparison with Other Alien Populations

**DOI:** 10.3390/insects11040235

**Published:** 2020-04-09

**Authors:** Daniel Carrillo, Luisa F. Cruz, Alexandra M. Revynthi, Rita E. Duncan, Gary R. Bauchan, Ronald Ochoa, Paul E. Kendra, Samuel J. Bolton

**Affiliations:** 1 Tropical Research and Education Center, University of Florida, Homestead, FL 33031, USA; luisafcruz@ufl.edu (L.F.C.); arevynthi@ufl.edu (A.M.R.); ritad@ufl.edu (R.E.D.); 2 Electron and Confocal Microscopy Unit, United States Department of Agriculture, Agricultural Research Service, Beltsville, MD 20705, USA; gary.bauchan@usda.gov; 3 Systematic Entomology Laboratory, United States Department of Agriculture, Agricultural Research Service, Beltsville, MD 20705, USA; ron.ochoa@usda.gov; 4 Subtropical Horticulture Research Station, Miami, United States Department of Agriculture, Agricultural Research Service, Miami, FL 33158, USA; paul.kendra@usda.gov; 5 Division of Plant Industry, Florida Department of Agriculture and Consumer Services, Gainesville, FL 32614, USA; Samuel.Bolton@FDACS.gov

**Keywords:** invasive mite species, erinea, *Litchi chinensis*, galls

## Abstract

The lychee erinose mite (LEM), *Aceria litchii* (Keifer) is a serious pest of lychee (*Litchi chinensis* Sonn.). LEM causes a type of gall called ‘erineum’ (abnormal felty growth of trichomes from the epidermis), where it feeds, reproduces and protects itself from biotic and abiotic adversities. In February of 2018, LEM was found in a commercial lychee orchard on Pine Island, Florida. Infestations were recorded on young leaves, stems, and inflorescences of approximately 30 young trees (1.5–3.0 yrs.) of three lychee varieties presenting abundant new growth. Although LEM is present in Hawaii, this mite is a prioritized quarantine pest in the continental USA and its territories. Florida LEM specimens showed small morphological differences from the original taxonomic descriptions of Keifer (1943) and Huang (2008). The observed differences are probably an artifact of the drawings in the original descriptions. Molecular comparisons were conducted on the DNA of LEM specimens from India, Hawaii, Brazil, Taiwan, Australia and Florida. The amplified COI fragment showed very low nucleotide variation among the locations and thus, could be used for accurate LEM identification. The ITS1 sequences and partial 5.8S fragments displayed no nucleotide differences for specimens from any of the locations except Australia. Consistent differences were observed in the ITS2 and 28S fragments. The ITS1-ITS2 concatenated phylogeny yielded two lineages, with Australia in one group and Hawaii, India, Brazil, Florida and Taiwan in another. Specimens from Taiwan and Florida present identical ITS and rDNA segments, suggesting a common origin; however, analysis of additional sequences is needed to confirm the origin of the Florida population.

## 1. Introduction

The Lychee Erinose Mite (LEM), *Aceria litchii* (Keifer) (Acari: Eriophyidae) is one of the most important pests of lychee trees (*Litchi chinensis* Sonn., Sapindaceae) in Asia. It occurs throughout India [1], Pakistan [2], Bangladesh [3], Thailand [4], China and Taiwan [5]. The mite has also been reported from Hawaii [6] and Australia [7]. More recently, LEM was found in Brazil [8], where it has spread to all major lychee producing areas and has caused an estimated 70%–80% yield reduction and a 20% increase in production costs [9]. An 80% yield reduction caused by LEM was also reported in India [10].

**Figure 1:**
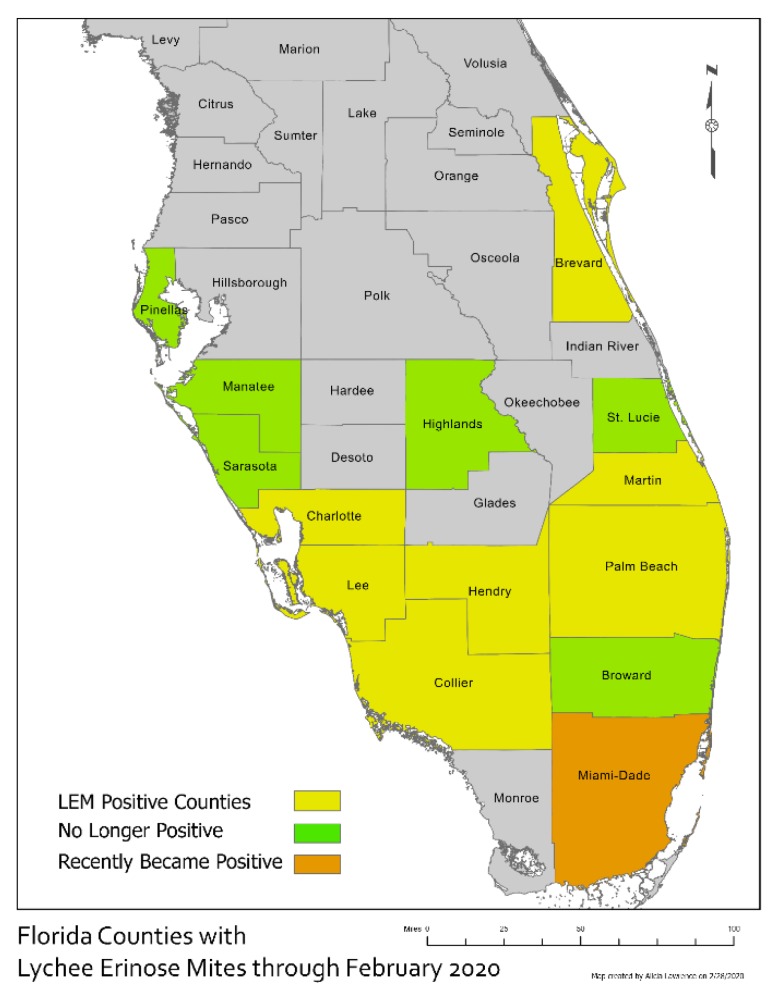
Current distribution of lychee erinose mite (LEM) (*Aceria litchii*) in Florida.

Although LEM is present in Hawaii [6], this mite is a prioritized quarantine pest in the continental United States of America and its territories [11]. Florida is the leading producer of lychee in the United States, followed by Hawaii and California [12], making LEM a pest of significant concern when it was detected in a 1.2 ha. commercial lychee orchard on Pine Island, Lee County, Florida in February 2018. Infestations were recorded on young leaves, stems, and inflorescences of approximately 30 young trees (1.5–3.0 yrs.) of three varieties (‘Mauritius’, ‘Hak Ip’, and ‘Sweet Heart’), presenting abundant new flush. Pine Island houses several nurseries that produce lychee tree propagative material and LEM-infested plants were transported to other Florida counties. Subsequent surveys conducted by the Florida Department of Agriculture and Consumer Services (FDACS) confirmed that Lee County was the epicenter of the LEM infestation, but small isolated LEM infestations were detected in 13 additional Florida counties (Brevard, Charlotte, Collier, Hendry, Highlands, Martin, Manatee, Palm Beach, Pinellas, Broward, Sarasota, Saint Lucie and Miami-Dade) (Figure 1). To prevent further spread of this major pest, FDACS imposed a quarantine on Lee County, prohibiting the movement of lychee fruit or plant parts (trees, leaves or stems) out of the county, and is currently implementing mitigation strategies in the other counties [13]. So far, isolated LEM infestations have been eradicated from six counties (Manatee, Broward, Highlands, Pinellas, Sarasota and Saint Lucie).

**Figure 2:**
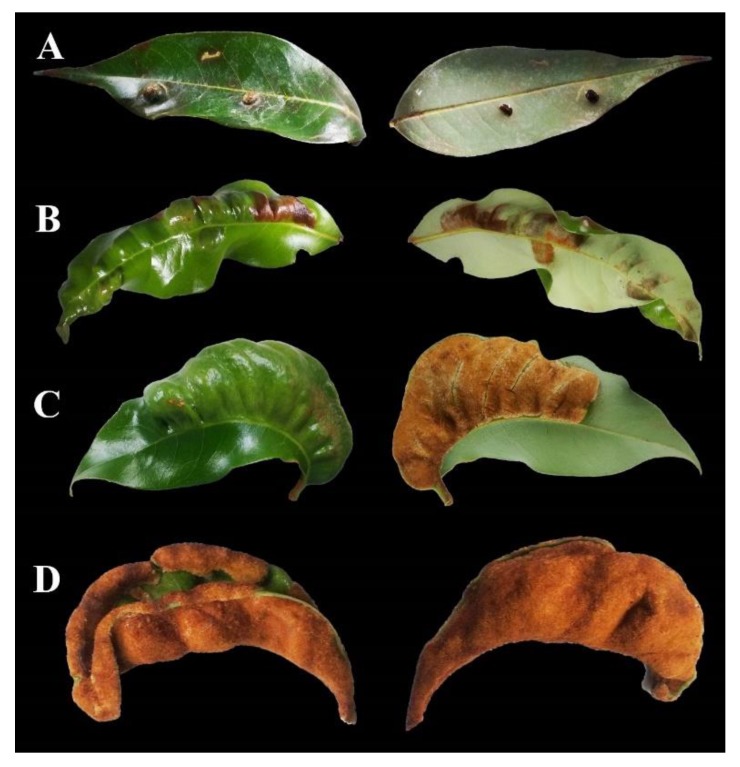
Erinea types associated with different levels of infestation of LEM-topside on left, underside on right (note that most of the topside of (**D**) is obscured because the leaf has folded). (**A**) Low level of infestation. (**B**) Moderate level of infestation. (**C**) High level of infestation. (**D**) Extreme level of infestation (topside of leaf with some erineum hairs).

As with other eriophyids, LEM uses a series of stylets to pierce and feed on leaf epidermal cells. Punctured cells often die, but surrounding epidermal cells undergo morphological alterations resulting in the hyperplasia of leaf trichomes, referred to as “erinea” [14]. How LEM induces the formation of erinea is unknown. The enlargement and excessive branching of trichomes provide mites with a favorable habitat along with protection from natural enemies and abiotic adversities. The relative degree of erineum maturity can be gauged from the color, thickness and density of the trichomes (Figure 2). Erinea can also develop on stems, flowers and fruit.

LEM prefers to feed on new flush, which they infest by dispersing ambulatorily on the plant [2,15]. For long-range dispersal, LEM is phoretic on honeybees during the blooming season and can also drift on air currents [16,17]. LEM lays its eggs in the erinea and development from egg to adult takes approximately 14 days [18]. Multiple, overlapping generations can occur over the course of a year and population growth seems to be favored by new growth on trees during moderately hot and dry periods. LEM is highly host-specific and is only known to attack lychee [19], with young trees being more susceptible. The main lychee varieties grown in Florida are ‘Mauritius’, ‘Brewster’ and ‘Sweetheart’, while several other varieties (19), including ‘Hak Ip’, ‘Bengal’, ‘Ohia’ and ‘Kaimana’, are cultivated on a smaller scale [20]. LEM infestations in Florida have been found in all major cultivars as well as on ‘Hak Ip’ and ‘Ohia’ (Carrillo and Revynthi, personal observations). There have been reports of longan (*Dinocarpus longan* Lour, Sapindaceae) serving as a host for LEM [5,21]. However, lychee and longan are often planted together in areas with high LEM pressure in Brazil and Florida and, so far, there is no other record of LEM infesting longan. Huang’s finding may be a case of dispersing LEM landing on a longan tree, a misidentification of the host plant (there are lychee–longan hybrids that could be mistaken for pure longan), or taxonomic confusion with the close mite relative *Aceria longana* Boczek and Knihinicki, which is specific to longan.

Proper identification is crucial for regulated pests. The identification of eriophyoids based exclusively on morphological characters requires very specific skills and relies on the availability of voucher specimens and accurate descriptions. The specimens detected in Florida resembled the descriptions of LEM by Keifer [6] and Huang [21]. However, there were noticeable differences between those descriptions and the Florida specimens. Unfortunately, Keifer’s original type specimens, housed at the Beltsville Agricultural Research Center (USDA), were unavailable for study due to their poor condition. Therefore, fresh specimens were collected from Hawaii, the type locality of LEM [6], for morphological comparison with the Florida specimens. In addition, DNA sequences widely used as molecular markers for other mite species were analyzed. These sequences were used to compare LEM populations from different parts of the world.

## 2. Materials and Methods

For morphological comparison, lychee erinose mites were freshly collected from Hawaii and Florida, preserved in 70% ethanol and then examined under Low-Temperature SEM (LTSEM), following the technique delineated by Bolton et al. [22]. Due to a poor state of preservation, the slide mounted type specimens of Keifer [6] were unavailable for comparison with fresh material.

DNA was extracted from ten individual mites from Hawaii, Florida, India, Brazil, Australia, and Taiwan, using the protocol reported by Carew et al. [23]. Amplification of the cytochrome oxidase subunit I (COI) was carried out with the primer and conditions reported by Navajas et al. [24].The internal transcribed spacer 1 (ITS1) along with the 5′ terminal region of 5.8S were amplified with the primer sets and conditions reported by Fenton et al. [25]. The internal transcribed spacer 2 (ITS2) along with the 5′ terminal region of the 28S nuclear ribosomal rDNA (28S) were amplified with the primer sets and conditions reported by Sonnenberg et al. [26] and Chetverikov et al. [27]. DNA was amplified on a nexus G×2 thermocycler (Eppendorf, Hamburg, AG) in reaction mixtures (25 µL) containing the following components: 12.5 µL Platinum SuperFi Green PCR master Mix (Invitrogen, Carlsbad, CA, USA) 0.4 µM each primer and 3 µL DNA template. PCR products were verified by gel electrophoresis. The amplicon size was 460 bp for COI, 500 bp for ITS1a and 1.6kb for ITS2. PCR products were purified using ExoSAP-IT (Affymetrix, Santa Clara, CA, USA) following the manufacturer’s protocols. Sanger sequencing was performed by Eurofins genomics (Louisville, KY, USA), amplification primers were also used as sequencing primers.

Geneious software, version 9.1.5 [28] and the Staden Package [29], were used to trim low-quality sequence end reads, create consensus gene sequences, and align sequences of the individual molecular markers of *A. litchii* from the different locations. The length of the resulting trimmed sequences used for the analyses was 492 bp for ITS1, 936 for ITS2 and 363 for COI. Sequences were deposited in GenBank (Table 1).

Phylogenies were generated for the individual COI and ITS1 sequence alignments and for the concatenated ITS1 and ITS2 segments by retrieving GenBank accessions of related Aceria species (Table S1). *Abacarus sacchari* Channabasavanna, 1966 was chosen as the outgroup.

The datasets of individual markers and the concatenated ITS1- ITS2 were aligned and trimmed to a similar length in Mega 7 software [30]. Phylogenies were generated under the maximum-likelihood method using 1000 bootstraps with Mega 7 software [30]. COI phylogeny was based on the Hasegawa–Kishino–Yano model [31] with discrete Gamma distribution to model evolutionary rate differences among sites, a total of 439 positions were used for this dataset. ITS1 phylogeny was based on Tamura 3-parameter model [32] with discrete Gamma distribution, a total of 509 positions were used in the final dataset. The concatenated ITS1-ITS2 was based on the Kimura 2-parameter model [33]; this dataset contained a total of 1170 positions.

Genetic diversity of the three molecular markers among the collection sites was described by estimating the number of haplotypes (COI) or alleles (ITS1 and ITS2), nucleotide diversity (π) and number of polymorphic segregating sites, these parameters were calculated in DnaSP version 6 software [34]. Estimations of pairwise divergency were computed using Kimura’s 2-parameter (K2P) distance model [33]. For COI, the first codon position was included and transition + transversion was chosen as the substitution type.

## 3. Results

**Figure 3:**
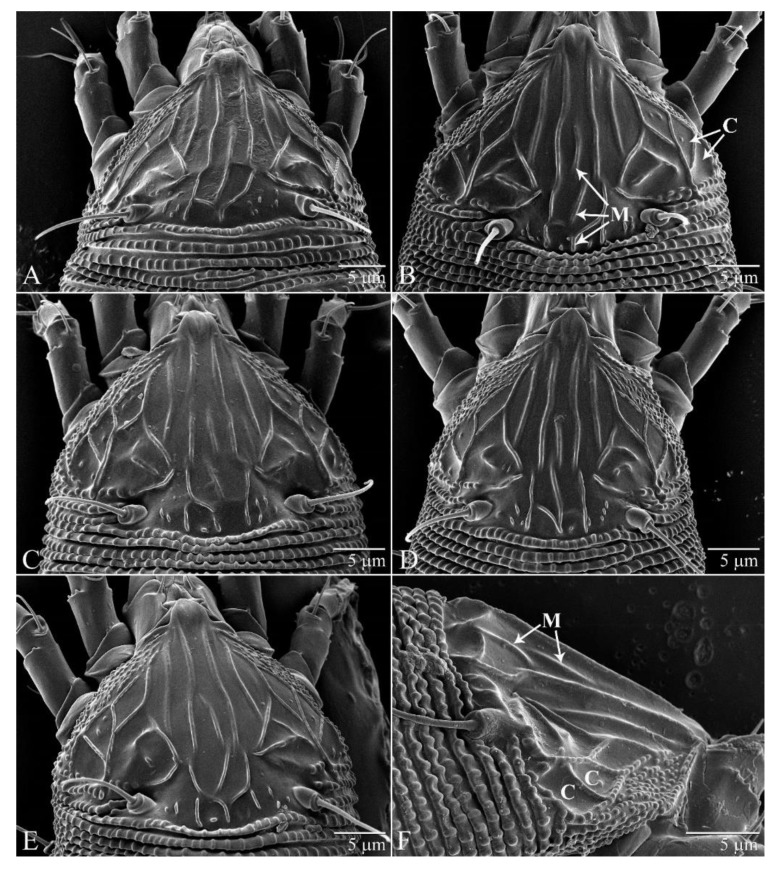
Variation of the prodorsum of LEM from Florida and Hawaii examined under Low-Temperature SEM (LTSEM), following the technique delineated by Bolton et al. [22] (**A**)–(**E**) Dorsal view of Florida specimens (unknown instars) (only (**B**) is labeled). Note that the median line is sometimes broken anteriorly and/or posteriorly. The arrangement of the ridges in the posteromedian region also varies dramatically so that the posterior part of the median line can run centrally, or curve left or right. (**F**) Lateral view of Hawaii specimen (unknown instar), showing the broken median line and the two additional lateral cells also observed in the Florida specimens. M = Median line; C = Cells missing from figure of Keifer [6]. Adults 110-135 μm long.

The specimens from Florida resemble the descriptions of LEM by Keifer [6] and Huang [21]. However, with respect to the prodorsum, there are three noticeable differences between the descriptions and the Florida specimens: (1) the median line of the Florida specimens is almost always broken, whereas it is unbroken in the drawn figures from the two descriptions by Keifer [6] and Huang [21]; (2) in the Florida specimens, the arrangement of ridges in the posteromedian region is typically asymmetrical and highly variable among specimens (Figure 3A–E), whereas it is symmetrical and varies little between the figures of Keifer [6] and Huang [21]; (3) the Florida specimens have three lateral cells on each side of the prodorsum (Figure 3B), whereas the Keifer description only appears to show one of those cells. LEM obtained from Hawaii were similar to the Florida specimens with respect to all three characters (Figure 3F).

**Figure 4:**
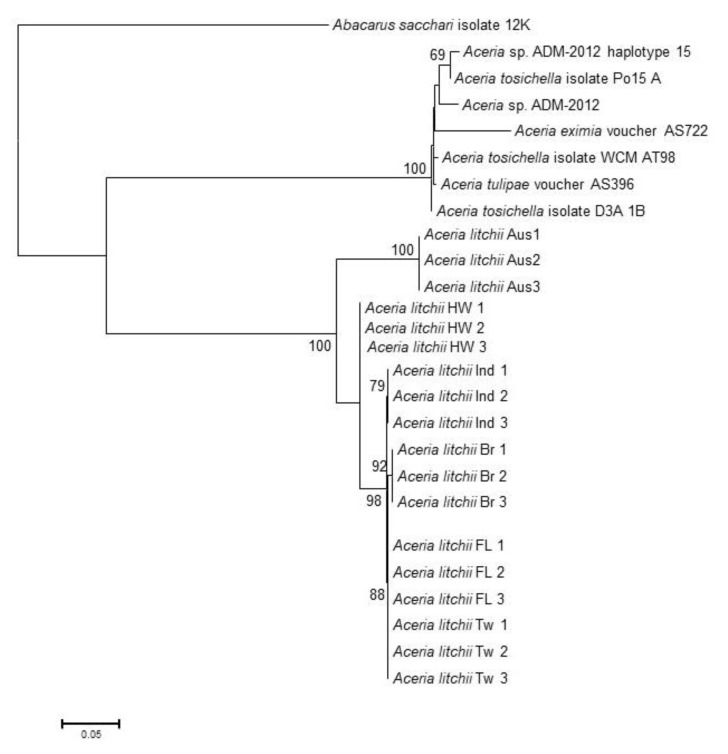
Phylogeny of *Aceria* species as inferred by Maximum Likelihood of the ITS1 and ITS2 concatenated sequences. Numbers presented above and below each node indicate maximum likelihood bootstrap values and Bayesian a posteriori probability for the corresponding inner branch.

All the individuals from each location had identical sequences for all the molecular markers. Analysis of the LEM-COI segments showed low sequence variability, as indicated by the lowest nucleotide diversity, number polymorphic segregating sites (Table 2), as well as the smallest genetic distances between the locations (Table 3). COI segments from Australia, Brazil and Hawaii shared 100% of identity, whereas Florida, Taiwan and India showed unique alleles. The ITS1 sequence and partial 5.8S fragment of the Florida LEM were identical to those of Hawaii, India, Brazil, and Taiwan. By contrast, Australia, with 62 polymorphic sites, shared 79.9% of identity with the rest of the locations (Table 2 and Table 3). The ITS2 and 28S partial sequences from Australia and Hawaii, as well as Taiwan and Florida, shared 100% sequence identity. This dataset showed the largest number of segregation sites distributed across the locations and the largest genetic distances among the molecular markers (Table 2 and Table 3). The phylogeny generated from the COI analysis resulted in a tree with two major clades. One clustered the LEM sequences from the six locations and the second clade contained all other *Aceria* spp. Due to the low intraspecific variation of the COI segment, no resolution was observed within the LEM clade (Figure S1). The analysis of the concatenated ITS1 and ITS2 provided resolution within the LEM clade (Figure 4), with Australia in the most basal position, followed by Hawaii, India and then Brazil, so that each of these locations is sister to a clade comprising the remaining locations, while Florida and Taiwan form the two most recently diverging locations.

## 4. Discussion

Differences between the Florida specimens and the descriptions of Keifer [6] and Huang [21] are probably an artifact of the drawings, which appear to simplify the detail and variation revealed in the LTSEM images.

The molecular analysis of the LEM specimens from different geographic areas suggests that the mitochondrial CO1 gene region, a standard genetic marker for a broad range of animals [35], is potentially appropriate for accurate identification of LEM. The COI sequence could be an important tool to identify LEM immature stages. Our results also suggest that the ITS1 and ITS2 regions are useful for studies on intraspecific variability. The ITS1 and ITS2 regions may be useful to trace specific LEM populations and identify introduction routes. This information may be useful to prevent reintroduction or further spread of this pest. The ITS2 and 28S sequences vary by geographic location. Divergence of the Australian population may have occurred earliest, resulting in the observed tree topology. Interestingly, LEMs from Taiwan and Florida seem to present identical genotypes, perhaps indicating the same origin. Further analysis is required to ascertain the origin of the Florida and Taiwan populations. We were unable to obtain specimens from Southern China, where lychee originates [36]. Future efforts to study the biogeography of LEM should include specimens from Southern China.

Lychee is a difficult crop for farmers in Florida because yield can vary greatly due to irregular flowering and poor fruiting retention [12]. The establishment of LEM in commercial orchards could become a major pest problem that could further reduce yields. It is unknown how LEM was introduced into Lee County, Florida. Considering the geographic distance between the known geographic range of LEM and Florida, the founders of the introduced populations must have been transported by human activities. The detection reported here is the third introduction of LEM in Florida. The first detection was in 1955 in a lychee grove located at Nokomis, Sarasota County. This introduction failed to establish and was likely eradicated through a combination of extensive pruning, acaricide treatments and extremely cold temperatures, which were reported in 1960 [37]. The second interception was in Coral Gables, Miami-Dade County in 1993; LEM infested containerized trees imported from China, which were destroyed to eradicate the pest [38]. The population recently detected on Pine Island has already spread to multiple Florida counties. Notably, the principal lychee commercial growing area in South Florida is, so far, free of LEM. Suppression or eradication of this current LEM invasion in Florida will depend largely on the strategic actions of regulatory agencies and industry to manage this pest.

Known management tools targeting LEM are restricted to cultural and chemical practices. Postharvest pruning, which includes eliminating and burning infested branches, is the main cultural control strategy against LEM [39,40]. Chemical control has been studied in Australia, China, and Brazil [7,14,39]. Several active ingredients have shown some efficacy on LEM [15,41]. The main management strategy focuses on protection of new flush after the postharvest sanitation through carefully timed acaricide applications [39,42]. Sprays of dimethoate or wettable sulfur at 2–3 week intervals, starting at bud emergence until leaves have hardened, provided protection of new flush against LEM in Australia [39]. In Hawaii, sulfur applications have been used to protect new flush against LEM [43]. A recent study indicates that post-harvest paraffinic oil dips have potential to disinfest lychee fruit from LEM, thereby minimizing risk of further pest spread in Florida [44].

Biological control might provide a long-term solution to managing this invasive mite. Several natural enemies have been reported in association with LEM in India [45], Australia [46,47], Brazil [42,48] and China [49]. However, predation on LEM has only been confirmed for a few species of phytoseiid mites, including *Amblyseius largoensis* (Muma) in China [50], and *Phytoseius intermedius* Evans and MacFarlane in Brazil [48]. In Florida, *A. largoensis*, *Euseius membrasicus*, *Hemycheyletia bakeri* and *Cheylotogenes ornatus* have been found to be associated with LEM. The mite pathogen *Hirsutella thompsonii* (Fischer) has also been reported to infect LEM in Brazil [51].

The State of Florida is conducting an eradication program for LEM [13]. If the eradication program is not successful, a comprehensive program for managing LEM will require: (1) detailed investigations on the biology and ecology of LEM, (2) implementation of effective regulatory actions to prevent movement of LEM infested material, (3) development of phytosanitary postharvest treatments, (4) determination of the susceptibility of lychee cultivars grown in neighboring areas, and (5) development of biological and chemical control programs against LEM.

## 5. Conclusions

The invasive LEM was recently found on Pine Island, Florida. Infestations have spread to several FL counties but have not reached the main commercial production area in South Florida. Initial taxonomic uncertainties were resolved through comparison of the Florida specimens with fresh specimens collected from Hawaii, the type locality. A molecular analysis of specimens from India, Hawaii, Brazil, Taiwan, Australia and Florida revealed that LEM from Australia had diverged earlier and that LEM from Taiwan and Florida may have a common origin. The methodologies used here can be used to accurately identify LEM and trace further movement of LEM populations. The impact of LEM on Florida’s lychee industry will depend on the success of an eradication program currently conducted by the state’s regulatory agency. Management programs specific for LEM are lacking in Florida.

## Figures and Tables

**Table 1:** Lychee erinose mite (*Aceria litchii*) specimens collected from different countries and used for DNA fragment sequence analysis. GenBank accession numbers for all analyzed sequences are provided.

Geographic Origin	Coordinates	Collector	Collection Date	GenBank Sequences		
				COI	ITS1-5.8	ITS2-28S 5’
**Australia**	**24.87 S 56.78 E**	**E. Dunn & J.J. Beard**	3-Apr-19	MT027817	MN594262	MN594268
Brazil	22.71 S 43.63 W	G.J de Moraes	25-Mar-19	MT027818	MN594263	MN594269
Florida	26.6 N 82.1 W	D. Carrillo & A.M Revynthi	20-Feb-18	MT027819	MN594264	MN594270
Hawaii	21.30 N 157.82 W	B. Azama & M. Ramadan	15-Mar-18	MT027820	MN594265	MN594271
India	22.94 N 88.53 E	K. Karmakar	25-Mar-19	MT027821	MN594266	MN594272
Taiwan	22.64 N 120.35 E	C.F. Hong	22-Apr-19	MT027822	MN594267	MN594273

**Table 2:** Sequence diversity estimates for *A. litchii.*

Molecular Marker	Number of Sites	No. of Alleles/Haplotypes	No. of Segregating Sites	Nucleotide Diversity Per Site (π)
ITS1	492	2	62	0.04665
ITS2	936	4	64	0.03874
COI	363	4	6	0.00724

**Table 3:** Pairwise K2P distances between the *A. litchii* from six locations.

	ITS1					ITS2					COI				
	FL	Au	Br	HW	Ind	FL	Au	Br	HW	Ind	FL	Au	Br	HW	Ind
Au	0.140					0.066					0.008				
Br	0.000	0.140				0.004	0.069				0.008	0.000			
HW	0.000	0.140	0.000			0.066	0.000	0.069			0.008	0.000	0.000		
Ind	0.000	0.140	0.000	0.000		0.009	0.067	0.009	0.067		0.017	0.008	0.008	0.008	
Tw	0.000	0.140	0.000	0.000	0.000	0.000	0.066	0.004	0.066	0.009	0.017	0.008	0.008	0.008	0.000

## Supplementary Materials

The following are available online at https://www.mdpi.com/2075-4450/11/4/235/s1, Figure S1: Phylogeny of *Aceria* species Cytochrome oxidase 1 by the Maximum Likelihood method, Figure S2: Phylogeny of Aceria species ITS1 region by the Maximum Likelihood method, Table S1: GenBank accession of sequences used in the phylogenetic analyses.
