# Supplementary material for: Detection of the Lychee Erinose Mite, Aceria litchii (Keifer) (Acari: Eriophyidae) in Florida, USA: A Comparison with Other Alien Populations
Source: Insects. 2020 Apr 9;11(4):235. doi: 10.3390/insects11040235 (PMC7240363; doi:10.3390/insects11040235)
Supplement: Supplementary file 1 [file insects-11-00235-s001.docx]

**
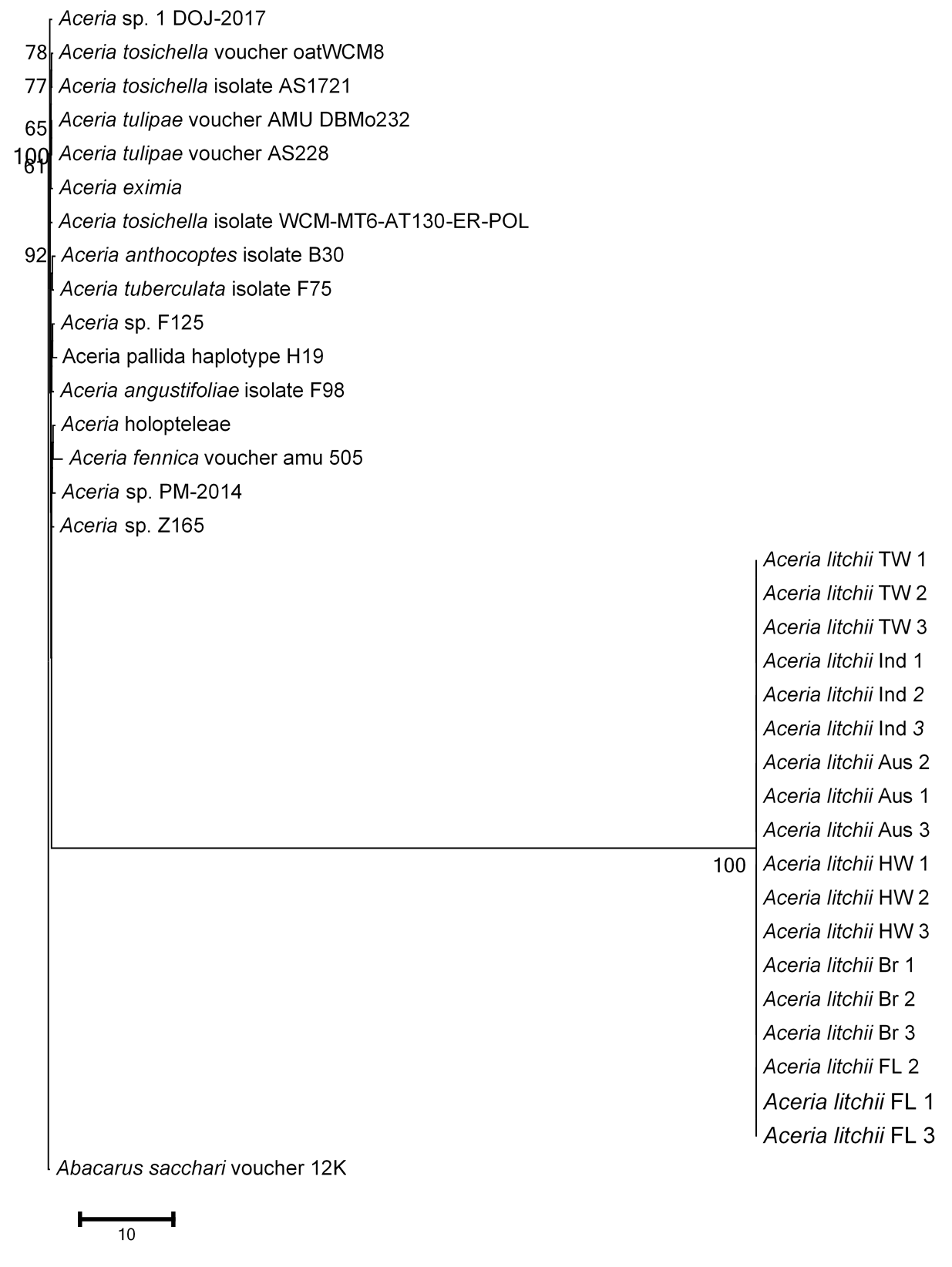
**

**Figure S1.** Phylogeny of *Aceria* species inferred by the Maximum Likelihood of the COI segment. Numbers above or below each node indicate maximum likelihood bootstrap values and Bayesian a posteriori probabilities for the corresponding inner branch.

**Figure S2.** Molecular Phylogenetic analysis by Maximum Likelihood of the ITS1 segment. Numbers above or below each node indicate maximum likelihood bootstrap values and Bayesian a posteriori probabilities for the corresponding inner branch.

*Aceria tosichella* isolate WCM AS1102

*Aceria tulipae*

*Aceria tosichella* isolate Kansas

*Aceria tosichella* isolate Montana

*Aceria tosichella* isolate South Dakota

*Aceria tulipae* isolate Oregon

*Aceria tulipae* isolate Ontario

*Aceria tulipae* isolate Nebraska

*Aceria tosichella* isolate WCM2b

*Aceria tosichella* isolate BRA503 3x

*Aceria* sp. B MC-2008

*Aceria* sp. ADM-2012 haplotype 13

Aceria eximia voucher AS722

*Aceria parapopuli* haplotype A

*Aceria parapopuli* clone OG1D-2a

*Aceria parapopuli* clone B23-1c

*Aceria parapopuli* clone ZMG19e

*Aceria parapopuli* clone HEN 3e

*Aceria parapopuli* clone L15b

*Aceria parapopuli* clone 18-34-3f2

*Aceria cajani*

*Aceria litchii* HW3

*Aceria litchii* HW2

*Aceria litchii* Br 3

*Aceria litchii* Br 2

*Aceria litchii* Br 1

*Aceria litchii* Ind 3

*Aceria litchii* Ind 2

*Aceria litchii* Ind 1

*Aceria litchii* Tw 3

*Aceria litchii* Tw 2

*Aceria litchii* Tw 1

*Aceria litchii* FL 3

*Aceria litchii* FL 2

*Aceria litchii* FL 1

*Aceria litchii* HW1

*Aceria litchii* Aus 3

*Aceria litchii* Aus 2

*Aceria litchii* Aus 1

*Abacarus sacchari* isolate 12K

81

99

84

98

95

86

98

95

99

0.2

**Table S1**. GenBank accession numbers of sequences used in the phylogenetic analyses

| Species | GenBank accession No | |
| --- | --- | --- |
| COI | | |
| *Aceria* sp. 1 DOJ-2017 | | MF374740.1 |
| *Aceria* sp. PM-2014 | | KM114210.1 |
| *Aceria* sp. Z165 | | KY888666.1 |
| *Aceria* sp. F125 | | KT070218.1 |
| *Aceria eximia* | | EF409415.1 |
| *Aceria pallida* haplotype H19 | | KR258856.1 |
| *Aceria tuberculata* isolate F75 | | KT070220.1 |
| *Aceria anthocoptes* isolate B30 | | KT070219.1 |
| *Aceria angustifoliae* isolate F98 | | KT070217.1 |
| *Aceria tulipae* voucher AMU DBMo232 | | KC907079.1 |
| *Aceria tulipae* voucher AS228 | | JF920096.1 |
| *Aceria fennica* voucher amu 505 | | KU315234.1 |
| *Aceria holopteleae* | | KJ872678.1 |
| *Aceria tosichella* isolate WCM-MT6-AT130-ER-POL | | KX430283.1 |
| *Aceria tosichella* isolate AS1721 | | MG194415.1 |
| *Aceria tosichella* voucher oatWCM8 | | JQ248924.1 |
| *Abacarus sacchari* voucher 12K | | KX892630.1 |
| ITS1 | | |
| *Aceria tosichella* isolate South Dakota | | JX087352.1 |
| *Aceria tosichella* isolate Montana | | JX087353.1 |
| *Aceria tosichella* isolate Kansas | | JX087354.1 |
| *Aceria tosichella* isolate WCM AS1102 | | KJ000671.1 |
| *Aceria tulipae* isolate Oregon | | JX087357.1 |
| *Aceria tulipae* isolate Nebraska | | JX087360.1 |
| *Aceria tulipae* isolate Ontario | | JX087361.1 |
| *Aceria tosichella* isolate WCM2b | | EU734724.1 |
| *Aceria* sp. ADM-2012 haplotype 1 | | JQ512784.1 |
| *Aceria* sp. B MC-2008 | | EU734727.1 |
| *Aceria eximia* voucher AS722 | | JF920113.1 |
| *Aceria parapopuli* haplotype A | | JF792213.1 |
| *Aceria parapopuli* clone HEN 3e | | EF641824.1 |
| *Aceria parapopuli* clone ZMG19e | | EF641835.1 |
| *Aceria parapopuli* clone 18-34-3f2 | | EF641814.1 |
| *Aceria parapopuli* clone L15b | | EF641828.1 |
| *Aceria parapopuli* clone B23-1c | | EF641817.1 |
| *Aceria parapopuli* clone OG1D-2a | | EF641829.1 |
| *Aceria tulipae* isolate Oregon | | JX087357.1 |
| *Aceria cajani* | | AJ251693.1 |
| ITS2-ITS1 | | |
| *Aceria tulipae* voucher AS396 | | JF920112.1 |
| *Aceria eximia* voucher AS722 | | JF920113.1 |
| *Aceria tosichella* isolate Po15 A | | JF960156.1 |
| *Aceria tosichella* isolate D3A 1B | | JF960160.1 |
| *Aceria tosichella* isolate WCM AT98 | | KJ000667.1 |
| *Aceria* sp. ADM-2012 haplotype 13 | | JQ512796.1 |
| *Abacarus sacchari* isolate 12 K | | KX855733.1 |
